# Stunting as a Risk Factor of Soil-Transmitted Helminthiasis in Children: A Literature Review

**DOI:** 10.1155/2022/8929025

**Published:** 2022-08-03

**Authors:** Nisa Fauziah, Muhammad Abdurrahman Ar-Rizqi, Sofia Hana, Nurul Mufliha Patahuddin, Ajib Diptyanusa

**Affiliations:** ^1^Parasitology Division, Department of Biomedical Sciences, Universitas Padjadjaran, Bandung, Indonesia; ^2^Advanced Biomedical Parasitology Laboratory, Faculty of Medicine, Universitas Padjadjaran, Bandung, Indonesia; ^3^Faculty of Medicine, Universitas Padjadjaran, Bandung, Indonesia; ^4^Department of Parasitology, Faculty of Medicine, Public Health and Nursing Universitas Gadjah Mada, Yogyakarta, Indonesia

## Abstract

As a high-burden neglected tropical disease, soil-transmitted helminth (STH) infections remain a major problem in the world, especially among children under five years of age. Since young children are at high risk of being infected, STH infection can have a long-term negative impact on their life, including impaired growth and development. Stunting, a form of malnutrition in young children, has been long assumed as one of the risk factors in acquiring the STH infections. However, the studies on STH infection in children under five with stunting have been lacking, resulting in poor identification of the risk. Accordingly, we collected and reviewed existing related research articles to provide an overview of STH infection in a susceptible population of stunted children under five years of age in terms of prevalence and risk factors. There were 17 studies included in this review related to infection with *Ascaris lumbricoides*, *Trichuris trichiura*, hookworm, and *Strongyloides stercoralis* from various countries. The prevalence of STH infection in stunted children ranged from 12.5% to 56.5%. Increased inflammatory markers and intestinal microbiota dysbiosis might have increased the intensity of STH infection in stunted children that caused impairment in the immune system. While the age from 2 to 5 years along with poor hygiene and sanitation has shown to be the most common risk factors of STH infections in stunted children; currently there are no studies that show direct results of stunting as a risk factor for STH infection. While stunting itself may affect the pathogenesis of STH infection, further research on stunting as a risk factor for STH infection is encouraged.

## 1. Introduction

Soil-transmitted helminthic (STH) infections caused by either *Ascaris lumbricoides*, *Trichuris trichiura*, hookworms, or *Strongyloidiasis stercoralis* are some of the neglected tropical diseases (NTDs) which affect about 1.5 billion people around the world [1]. The burden of ascariasis, trichuriasis, and hookworm diseases is reported to be the highest in Asia, Africa, and Latin America. In 2017, STH infections resulted in approximately 1.6 million years lived with disability (YLD) [2, 3]. Among these regions, Southeast Asian countries account for the highest burden of STH infection [4]. While Indonesia is generally thought to have a high prevalence of STH infections, only limited data are currently published.

One of the high-risk groups for acquiring STH infections are children under five years of age [1]. In this particular population, the rates of morbidity and mortality due to infection of STH are higher than in other populations, since the disease may progress to complications, including intestinal obstruction and necrosis, and rectal prolapse [5, 6]. Long-term impact of STH infections in young children should also be of concern, because it may cause developmental and growth disorders that can cause cognitive decline due to impairment of the brain's maturation process, affecting future learning and education [7–9]. There are several factors that make young children more susceptible to the STH infections, one of which is malnutrition [10–14]. Stunting can cause children under five years of age to be more susceptible to infection, since it can weaken the immune system indirectly through changes in the structure and function of the intestinal mucosa as well as changes in intestinal microbes resulting in increased susceptibility to infections [14, 15].

While recent published studies mainly focused on STH infections in school-aged children, research on the relationship between stunting and STH infections in young children has been lacking. Considering the potential risk of mortality and morbidity of STH infection in young children, the estimation of disease burden and risk factors of contracting such infection should be studied in detail. The current review emphasizes the epidemiology and symptoms of STH infection in stunted children under five years of age. Additionally, we aimed to provide information on the risk factors of STH infection in the same population, with focus on stunting. We believe that current review results can provide supporting data for further identification of high-risk populations to be the target of STH prevention programs.

## 2. Methods

### 2.1. Literature Search Strategy

This study performed article searches on electronic databases: PubMed/MEDLINE, Science Direct, and Google Scholar. Keywords were defined using PICO method in literature searching strategy (Table 1). The keywords include stunting, malnutrition, undernutrition, STH, helminth, *Ascaris*, *Trichuris*, hookworm, and *Strongyloides*. Boolean terms and truncation were also used during the literature search. In addition to the mentioned methods, the current study also used a hand-searching and snowball approach to include more studies. Searches were limited to articles written in either English or Bahasa Indonesia that were published from 2010 to 2020. Articles were screened for the title and abstract according to eligibility criteria. After duplicates were removed, eligible articles underwent full-text review.

### 2.2. Selection Criteria

**Figure 1:**
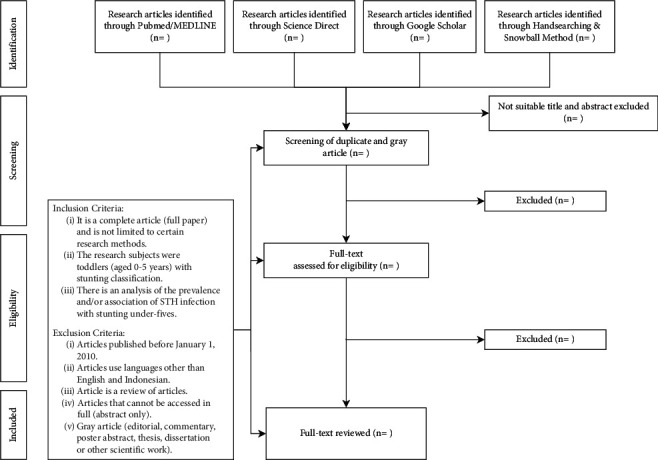
Literature review strategy.

All literature works were assessed for eligibility by the authors. The inclusion criteria used in the current review were the articles indexed in PubMed/MEDLINE or Science Direct or Google Scholar, and a full research was conducted in study population of stunted children under five years of age. The articles were excluded from the analysis if one of the following exclusion criteria were met: articles published before 2010, articles written in other than English or Bahasa Indonesia, a review of articles, abstract only articles, and gray articles (Figure 1).

### 2.3. Data Extraction

The data from various articles were extracted and presented in tabular form. The extracted data included: authors, year of publication, study location, population age, total population, research methods, disease, prevalence of STH infection, prevalence of STH infection in stunted children under five years of age, clinical manifestations, laboratory findings, risk factors, and the effect size of the risk factor. When the relevant data were unavailable or unobtainable from the article, they were written as “not available” (N/A).

## 3. Results

### 3.1. Overview

**Figure 2:**
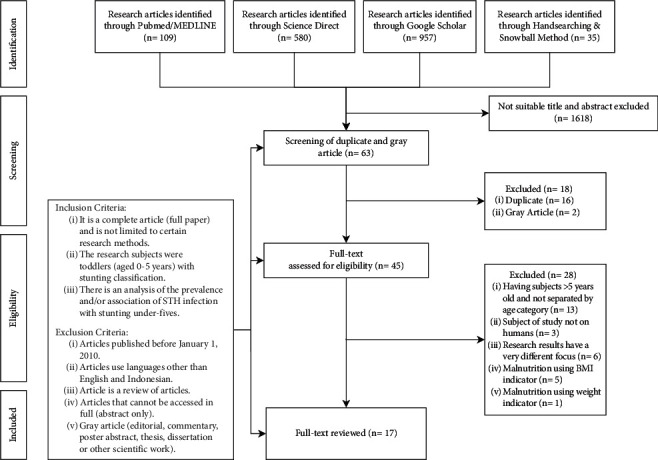
Study flow.

The current review included 17 articles from 1,681 articles found in various online databases: PubMed/MEDLINE (https://pubmed.ncbi.nlm.nih.gov), ScienceDirect (https://www.sciencedirect.com), and Google Scholar (https://scholar.google.com). Articles were also retrieved from these databases using snowball and hand-searching methods. As many as 63 articles passed the first screening, and after rescreening for duplicates, 45 articles remained for full-text review. A total of 28 studies were excluded, leaving 17 research articles with various designs included in the final analysis. Reasons for exclusion include older population (13 articles), nonhuman subjects (3 articles), having a very different focus (6 articles), and malnutrition-used indicators other than height and weight (6 articles). Disease proportion and epidemiological characteristics were extracted from the 17 articles, while clinical manifestations were retrieved from 8 articles, and risk factors were taken from 5 articles. The flow of the literature search strategy is described in Figure 2.

### 3.2. Epidemiology

Almost all articles discussed the prevalence of the three types of STH infection, namely, ascariasis (*n* = 15/17), trichuriasis (*n* = 14/17), and hookworm infection (*n* = 13/17). Strongyloidiasis was only discussed in four articles, with one in South America and the other three in Africa. Two articles did not provide details on the prevalence of helminthiasis-causing species. The largest proportion of the studies identified was conducted on the African continent (*n* = 7/17). The other studies were conducted in South America (*n* = 3/17), North America (*n* = 3/17), and Asia (*n* = 4/17). The general prevalence of STH infection in children under five obtained from several research articles reviewed in this study ranged from 4.3% to 62.7%. The prevalence of ascariasis ranged from 9.7% to 47.1%, while the prevalence of trichuriasis ranged from 0.5% to 24.0%, and hookworm infection prevalence ranged from 0% to 41.8%. The prevalence of *Strongyloides stercoralis* infection ranged from 0% to 4.8%. Overall, the STH infection prevalence in stunted children under five years of age ranged from 12.5% to 56.5% (Table 2).

### 3.3. Clinical and Laboratory Findings

The STH infections have a wide range of signs and symptoms, particularly gastrointestinal symptoms. The articles in our review mentioned clinical findings of both monoinfection and mixed infection of the helminth species in children. The most common clinical manifestations reported were abdominal bloating, diarrhea, and symptoms of iron deficiency anemia including pale ocular conjunctiva. Additionally, laboratory findings of STH infections include increased inflammatory markers (alpha-1 antitrypsin, IgA, and myeloperoxidase) and decreased ferritin. The clinical and laboratory findings of STH infections in children are described in Tables 3 and 4.

### 3.4. Risk Factors

Five of the seventeen (*n* = 5/17) articles in this study mentioned risk factors for STH infection in children under five years of age. An association between age factors, particularly the age of two to five years with STH infection, was also mentioned in two articles. Economic factors indicated there was an association with STH infection in two articles. Meanwhile, malnutrition was found to have no significant association with STH infection only in one article (Table 5).

## 4. Discussion

### 4.1. Prevalent STH Infection in Children under Five Years of Age

The current review revealed that the prevalence of STH infection in children under five years of age is still high, especially in stunted children. Multiple infections of more than one helminth species have been reported in this review as well. However, research on the population of stunted children under the age of five is still very little done. This should be a serious concern because according to the STH control program from the World Health Organization, by 2030 it is targeted to have a decrease in the prevalence and a decrease in morbidity from STH infection in the population of children under five years of age. Thereby, surveys or research should be held regularly in endemic countries [34]. The high prevalence of STH infection in stunted children has been estimated to occur due to multiple factors, one of which is age. The immune system in younger children has not yet reached maturity, resulting in their high vulnerability to infections [35]. However, one study showed that in younger children, particularly those during the breastfeeding period, their immune system is strengthened by the presence of immunoglobulins in breast milk [36]. This is in line with the findings in the study of Gyorkos et al. in 2011, which found a difference in prevalence between the age groups of 7–9 months and 12–14 months, where it was stated that the 12- to 14-months age group had a higher prevalence. Toddlers tend to be more at risk than infants as they are more active and are more exposed to different environment, which increases the risk of becoming infected with STH [17].

Three studies on the South American continent reported the prevalence of ascariasis in the population of children under five years of age ranging from 9.7% to 14.2% [17, 23, 24]. This is not much different from the previous systematic review study, which stated that the estimated prevalence of ascariasis in South America was around 15.6% [37]. Studies on the North American continent conducted in Mexico and Panama stated that the prevalence of ascariasis ranged from 20% to 33.6%. This was higher than previous studies, stating that the prevalence of ascariasis in Mexico in the population of children aged 1–4 years was only about 5.3% [19, 20, 30, 38]. The high prevalence of ascariasis in two studies in Mexico is considered to be due to a large number of local resident houses with dirt floors accompanied by poor hygiene, thereby facilitating the transmission of ascariasis [19, 30]. On the other hand, a study in Panama estimated that this is due to low maternal education and household factors such as the presence or absence of access to clean water and the absence of tiles on the floor [20]. The low level of education and employment of mothers can affect the vulnerability of children under five years of age to be infected with STH because these children are still dependent on those who provide care. This involves the essential food hygiene during preparation and consumption, skills which would have been lacking in less educated mothers [11, 39]. However, the study by Cabada et al. in the same population age found that the prevalence of ascariasis was below 10% [23]. This difference can be caused by the different geographical condition of the study locations and the mass deworming therapy in the population two weeks before taking the sample [23].

In this review, all studies on the African continent were conducted in sub-Saharan Africa. Four of the seven (*n* = 4/7) studies in Africa stated that the prevalence of STH infection in children under five years of age ranged from 10% to 14.7% [26, 27, 31–33]. This prevalence was not much different from the previous review, where the majority of the study population was school-age children. The results stated that the estimated prevalence of ascariasis in sub-Saharan Africa was around 13.6% [4]. Two other studies in Africa found a higher prevalence, namely, by Suchdev et al. in 2014 and Adeniran et al. in 2017, with 24.1% and 47.1%, respectively [22, 25]. A study conducted by Adeniran et al. in 2017 in Nigeria found a very high prevalence of ascariasis in children under five years of age, presumably due to the lack of behavior in washing hands with soap after defecation and before eating when toddlers play, and the habit of removing footwear when playing outside [25]. Nigeria is also known to be the most prevalent sub-Saharan country for STH infections [25, 40]. Meanwhile, three studies on the Asian continent, all conducted in Southeast Asia, stated that the prevalence of ascariasis in children under five ranged from 19.4% to 27.4% [18, 21, 28]. This is also not much different from the previous systematic review study, which said the prevalence of ascariasis in Southeast Asia was estimated to be around 25%, but the prevalence was in the school-age population [41].

The majority of studies in this review found the prevalence of trichuriasis below 10%. Eight of the fourteen (*n* = 8/14) studies that mentioned the prevalence of trichuriasis even founded that the prevalence of trichuriasis in children under five years was below 5% [19–21, 24, 26, 27, 30, 33]. This prevalence is similar to the previous review in which the majority of the studies in the review were in the school-age child population, which stated that the global prevalence of trichuriasis was around 8.3% [4]. Four studies found the prevalence of trichuriasis above 10%, namely, the study by Gyorkos et al. in 2011 with 22.3%, Kounnavong et al. in 2011 with 10.9%, Suchdev et al. in 2014 with 24%, and Cabada et al. in 2014 with 16.1% [17, 18, 22, 23]. These results indicate that the population of children under five years old still needs to be a serious concern because the prevalence of *Trichuris trichiura* infection in some places is still high.

Meanwhile, this review also shows that most studies found that the prevalence of hookworm infection and strongyloidiasis in children under five years of age is below 10%. In fact, all studies that mention the prevalence of strongyloidiasis find the prevalence below 5% [23, 25, 27, 32]. A very high prevalence of hookworm infection was found in a study conducted in Laos, which was around 41.8% [21]. Meanwhile, another study in Laos on the subjects of children aged 12–59 months found hookworm infection prevalence was around 10.9% [18]. Other studies conducted in Laos have also demonstrated similarly high prevalence of hookworm infection [42, 43]. This might have been due to poor knowledge of disease transmission, shown by local population who were doing daily activities barefoot, increasing the risk of infection [42]. Additionally, poor hygiene and sanitation practices in these populations augmented the possibility of disease transmission [42, 43]. Another factor contributing to the high prevalence in this study was that the deworming treatment program had not yet started at the time of data collection [21].

Prevalence rates of STH infections in stunted children and in children with normal nutritional status showed varying results in current review. One study showed no significant difference in prevalence between stunted children under five years old infected with STH (52.5%) and the overall prevalence of STH in children under five (54.1%) in Kenya [22]. On the other hand, a study conducted in Ethiopia in stunted children aged 6 months to 5 years demonstrated statistically different results, showing prevalence of 56.5% compared to that of normal children (26.3%) [32]. This could be due to the different methods in the two studies, which in the study by Suchdev et al. in 2014, only 7% of stool samples were reread for quality control, while in the study by Yoseph and Beyene in 2020, all samples were rechecked by blinding the examiner, which can increase the accuracy of the examination results [22, 32]. There were other studies conducted in Ethiopia and with a similar population [26, 33]. Both studies also have a similar prevalence of STH infection in children under five, namely 16.7% (*n* = 65/378) and 12.2% (*n* = 26/212), respectively. The study by Aiemjoy et al. in 2017 has data on the prevalence of STH infection in stunted toddlers, but there was no significant difference in the overall prevalence of STH infection in toddlers because it is estimated that the number of stunted subjects under five years old was small. The study by Jimenez et al. in 2013 stated that the prevalence of ascariasis in stunted children under five years of age was 31.4%, while the study by Jimenez et al. in 2019 found an increased prevalence of 51.3% [26, 30]. This difference is possibly due to the difference in population density between the two studies, where the study by Jimenez et al. in 2019 was conducted in Oxchuc, which has a higher population density than the study location by Jimenez et al. in 2013 [44]. High population density is known to be associated with an increased prevalence of STH [45].

Research by Ulayya et al. in 2018 conducted in Jombang Village, Indonesia, stated that the prevalence of children under five years old infected with STH was 6% (*n* = 3/50), while the prevalence of stunting in children under five years old was 26% (*n* = 13/50) [29]. The study did not mention the prevalence of STH infection in stunted children under five. The study by Huus et al. in 2020 in Madagascar and the Central African Republic had the same subjects as the study by Ulayya et al. in 2018, who were 2–5 years old, and also did not specify the prevalence of infection per STH species, but there was a significant difference in the prevalence of STH infection in children under five. Research by Huus et al. in 2020 stated that the prevalence of STH infection in children under five was 56% (*n* = 70/125) [31]. The study by Ulayya et al. 2018 is estimated to have a low prevalence due to the small number of subjects and different methods of diagnosing STH infection from other studies based on symptoms, not microscopic examination. This can affect the accuracy of the diagnosis of STH infection. The studies on STH infection in children under five years of age in Indonesia have been poorly reported. Studies were mostly focused on school-age children, which generally did not concern stunting condition in the population. This paucity of research must be a serious concern as Indonesia is the main contributor to the prevalence of STH infections in Southeast Asia. Childhood infection of STH may cause long-term growth impacts and may lead to a negative impact on future education.

### 4.2. Manifestations of STH Infection

Current review revealed only few studies mentioning the clinical manifestations of STH infections in stunted children [21, 22]. These studies revealed similar findings of STH infections in otherwise healthy children, including abdominal pain and bloating, diarrhea, and signs of anemia. However, abdominal bloating, occasional episodes of diarrhea, and pale conjunctivae were found to be more likely to occur in stunted children [21, 22]. The presence of malnutrition such as stunting may affect the general susceptibility of STH infection in this population. Stunting or chronic malnutrition can cause changes in the intestines, such as a reduction in the size of the villi and reduced immune cells that protect the intestines [46]. This can lead to a reduction in the barrier function of intestinal epithelial cells against foreign objects to enter deeper intestinal tissues and reduced protection from immune cells if there are unwanted foreign bodies that invade the intestinal tissue. Another condition that can happen is the increase in intestinal permeability (“leaky gut” syndrome), causing suboptimal nutrient absorption [47, 48]. Increased permeability can be a sign of damage to the intestinal tissue. These findings support the results mentioned in a study by Garzón et al. in 2017 that there was a significant addition of the intestinal inflammatory biomarker Alpha 1-Anti-Trypsin (A1AT) in stunting children under five years of age, which was increased by 50% [27]. Goblet cells in the intestines that should be stimulated to develop due to signals from immune cells during helminth infection were also found to be reduced in protein malnutrition compared to normal in the study on Wistar rats, which may result in reduced effectiveness of the expulsion mechanism of parasitic worms [46]. An increased proportion of IgA-coated bacteria was also found in stunting children in the study conducted by Huus et al. in 2020. The increase in IgA-coated bacteria is a marker of bacterial dysbiosis in the intestines, which usually occurs in conditions of intestinal inflammation [49, 50]. Intestinal bacterial dysbiosis, an imbalance of the normal gut microbiota, can interfere with the normal microbial protection function against infection in the intestine [51–54]. Therefore, this can make a person more susceptible to infections in the intestines, including intestinal parasites and STH infections.

In addition to changes in the intestinal structure, there are also changes in the immune system in people with a chronic malnutrition condition. It is found that there is an association between protein malnutrition and impaired B cells differentiation status in a study by Rho et al. in 2017. There was a lower ratio of immature B cells in mice with protein malnutrition, indicating impaired B cells differentiation status [55]. The activation of B cells that function in the production of antibodies, if disturbed, can cause the reduction of the body's ability to fight parasitic worms. Mast cells were also found to be significantly decreased in Wistar rats with malnutrition compared to those with normal nutritional status [46]. Mast cells are essential in triggering type 2 immunity in helminth infections through *T* helper 2 cells (Th2) activation [56, 57]. Th2 also produce IL-4, which functions to activate antibody class shifts on B cells to IgE, which is important for immunity to helminths [58]. In malnourished individuals, diminished type 2 immunity has been reported, resulted in slower parasite clearance and higher tendency to exacerbation of manifestations [46, 59]. These findings above can cause stunted children under five years of age to be more susceptible to STH infection. The intensity of STH infection in stunted children can also be more severe, which was identified in a study showing higher infection intensity in stunted children, reflected from number of eggs of *Ascaris lumbricoides* and hookworms per Gram in the feces [20]. Meanwhile, Gyorkos et al. in 2011 found the average number of *Ascaris lumbricoides*, *Trichuris trichiura*, and hookworm eggs per Gram in feces was higher in children of 12–14 months old compared to those of 7–9 months old [17]. Findings in the average number of eggs per Gram were almost the same in *Ascaris lumbricoides* and hookworm infections in toddlers aged 12-13 were also found in the study by Joseph et al. in 2014 [24]. These results indicate that the age factor may also play a role in determining the intensity of infection because older toddlers are more active to explore their environment and are more at risk for infection, including helminth infection.

In a study conducted by Halpenny et al. in 2013, hookworm reinfection was also reported to remain high in stunted children under five years of age compared to normal children despite being given a single dose of the antihelminthic drug, albendazole [20]. A possible mechanism to explain these findings is interference in the regulation of memory cells and proliferation of immune cells, as in a study by Iyer et al. in 2012 that found there could be a decrease in the proliferation and number of CD8^+^ memory T cells in malnourished test animals [60]. The study by Cabada et al. in 2014 stated no significant association between stunting toddlers and STH infection, but the prevalence of helminth infections was still high even after two weeks of mass treatment before taking stool specimens. This result is thought to be due to the effect of poor nutrition on effectiveness. Treatment due to the prevalence of stunting in the population 1–10 years of study is very high (up to 70%) [23].

### 4.3. Risk Factors of STH Infections in Children

**Figure 3:**
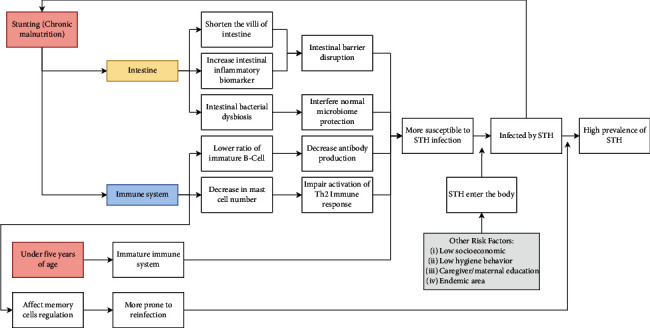
Summary of the possible mechanisms of stunting and under five years of age as a risk factor of STH infection (and reinfection), and other risk factors facilitating the increase of susceptibility of STH entry.

The study by Jimenez et al. in 2019 found that there was a significant association between *Ascaris lumbricoides* infection and stunting (OR = 9.81; *P*value <0.001) [30]. The same result was also found by Yoseph and Beyene in 2020, which stated an association between *Ascaris lumbricoides*, *Trichuris trichiura*, and hookworm infection with stunting [32]. However, Cabada et al. in 2014 found no significant association between stunting and STH infection in children under five [23]. Cabada et al. in 2014 conducted a study on 290 people, and 62 of them were toddlers. The low prevalence of infection or stunting under five in that study could be the cause of the insignificant statistical test results. In addition to stunting itself, which can be a risk factor because of its effect on immunity and the finding of an association with STH infection, the age factor is also important to be considered as a risk factor for STH infection. The immune system in children under five years old has not yet reached the same level of maturity as adults and is still assisted by antibodies from the mother in circulation and breast milk, and it is known that WHO recommends breastfeeding infants until up to two years of age [35, 61]. This is in line with the findings of the association of high STH infections with children aged 2–5 years in the study by Jimenez et al. in 2013 and Kounnavong et al. in 2011 [18, 19]. Breast milk components such as IgA antibodies are important for the maturation of the intestinal immune system and help fight infection, and the cellular components of breast milk are said to provide long-term resistance to worms in infants [36]. IgA is also said to have a protective factor against helminth infection because there is a negative correlation between IgA with the level of helminth infection in a study conducted on forest rats [62]. Socioeconomic status was also stated to have an association with STH infection. In a study by Kounnavong et al. in 2011, it was stated that toddlers with a high socioeconomic level had a 39% reduced risk of developing *Ascaris lumbricoides* (OR = 0.61; *P* value = 0.043) [18]. Yoseph and Beyene in 2020 also found that children under five living in low-income homes were 1.04 times more likely to develop STH infection, but this result was not significant. Other social factors were also mentioned, such as not wearing shoes and large family size as well as hygiene factors such as consuming raw vegetables and the absence of sanitation facilities. These other risk factors for STH infection make children more than twice as likely to be infected with STH [32]. Consumption of raw vegetables is known to increase the risk of exposure to STH eggs if they are not appropriately cleaned due to the possibility of contamination of STH eggs from the soil [63]. Not wearing shoes can also increase the risk of infection with STH that penetrates the skin to infect humans. Summary of potential mechanisms of STH infection and malnutrition in children under five years is depicted in Figure 3.

### 4.4. Study Limitations

There are several limitations involved in this review. First, the number of available literature on the topic was lacking, which can affect the overall results. Second, most of the studies reviewed in the current review were conducted in African countries, so the conclusion cannot be generalized to the overall population. Lastly, the included studies used a cross-sectional design that cannot clearly describe any cause-and-effect relationship between variables. Despite the limitations, the current review highlighted the high prevalence of STH infections in children under five years of age, which is a high-risk population that should receive more attention from the government to prevent the risk factors for STH infections. This study also emphasizes the low availability of studies in the population of stunted children under five years of age, thus providing an opportunity for the continuation of further studies in this vulnerable population. The current review also shows the possibility of how the mechanisms of stunting can lead to increased vulnerability in the population of children under five years of age. The current study results can be the basis for stunting prevention strategies as well as informing the importance of efforts toward prevention of STH infections in the population by controlling the identified risk factors.

## 5. Conclusions

The prevalence of STH infection in toddlers is still high, especially in stunted children under five years old. The common clinical manifestations of STH infection in children are gastrointestinal symptoms and signs of anemia. The severity of STH infections in stunted children was found to be different from children with normal nutritional status. Socioeconomic status, age, and hygiene factors also need to be considered as the contributing causes of the increased susceptibility or risk factors of STH infection. Although the pathogenesis is not fully clear, stunted children may have higher risk of getting STH infection. Current review results should aid direction of further research on this topic.

## Figures and Tables

**Table 1:** The PICO method to define keywords for searching strategy.

Population	Children under five years of age
Intervention/Exposure	Stunted children (according to the World Health Organization (WHO) Child Growth Standards) [16].
Control	Children with normal nutritional status
Outcome	STH infection caused by either *Ascaris lumbricoides*, *Trichuris trichiura*, hookworm (*Ancylostoma duodenale*, *Necator americanus*), or *Strongyloides stercoralis*

**Table 2:** Summary of studies in general and studies identifying proportion of STH infections in children under five.

Author (Year)	Study Location	Research Method	Number of Samples	Population Age	Disease	Prevalence	Stunting Prevalence	Ref
Gyorkos et al. (2011)	Peru	Cross sectional	164	7–9 months	Ascariasis	3/164 (1.8%)	25.9%	[17]
					Trichuriasis	2/164 (1.2%)		
					Hookworm infection	2/164 (1.2%)		
			185	12–14 months	Ascariasis	23/185 (12.4%)		
					Trichuriasis	39/185 (21.1%)		
					Hookworm infection	1/185 (0.5%)		

Kounnavong et al. (2011)	Laos	Cross sectional	570	12–59 months	Ascariasis	156/570 (27.4%)	48.9%	[18]
					Trichuriasis	62/570 (10.9%)		
					Hookworm infection	62/570 (10.9%)		

Jimenez et al. (2013)	Mexico	Cross sectional	250	0–5 years	Ascariasis	84/250 (33.6%)	60%	[19]
					Trichuriasis	3/250 (1.2%)		
					Ascariasis and trichuriasis	6/250 (2.4%)		

Halpenny et al. (2013)	Panama	RCT (randomized controlled trial)	356	0–48 months	Ascariasis	71/356 (20%)	72%	[20]
					Trichuriasis	4/356 (1%)		
					Hookworm infection	18/356 (5%)		

Sayasone et al. (2014)	Laos	Cross sectional	464	6 months–5 years	Ascariasis	90/464 (19.4%)	46.3%	[21]
					Trichuriasis	37/464 (8%)		
					Hookworm infection	194/464 (41.8%)		

Suchdev et al. (2014)	Kenya	Cross sectional	205	6 months—5years	Ascariasis	50/205 (24.1%)	29.7%	[22]
					Trichuriasis	47/205 (24%)		
					Hookworm infection	0%		

Cabada et al. (2014)	Peru	Cross sectional	62	0–5years	Ascariasis	6/62 (9.7%)	70.7% (in 0–10 years old population)	[23]
					Trichuriasis	10/62 (16.1%)		
					Hookworm infection	5/62 (8.1%)		
					Strongyloidiasis	3/62 (4.8%)		

Joseph et al. (2014)	Peru	Cross sectional	1760	12–13 months	Ascariasis	185/1760 (10.5%)	24.2%	[24]
					Trichuriasis	48/1760 (2.7%)		
					Hookworm infection	6/1760 (0.3%)		
					Any STH	219/1760 (12.4%)		

Adeniran et al. (2017)	Nigeria	Cross sectional	138	0–5 years	Ascariasis	65/138 (47.1%)	39.5% (in 0–71 months old population)	[25]
					Trichuriasis	7/138 (4.1%)		
					Hookworm infection	50/138 (36.2%)		
					Strongyloidiasis	5/138 (3.6%)		

Aiemjoy et al. (2017)	Ethiopia	Cross sectional	212	0–5 years	Ascariasis	23/212 (10.8%)	12.4%	[26]
					Trichuriasis	3/212 (1.4%)		
					Hookworm infection	0%		

Garzón et al. (2017)	Sao Tome and Principe Republic	Prospective cohort (until 24 months old)	80	28 days	Ascariasis	8/80 (10%)	23% (at the end of the study)	[27]
					Trichuriasis	2/80 (2.5%)		
					Hookworm infection	0%		
					Strongyloidiasis	0%		
					Ascariasis + giardiasis	4/80 (5%)		
					Trichuriasis + giardiasis	2/80 (2.5%)		
					Trichuriasis + ascariasis + giardiasis	1/80 (1.3%)		
					Cryptosporidium spp. + ascariasis	2/80 (2.5%)		
					Cryptosporidium spp. + trichuriasis	1/80 (1.3%)		

Nery et al. (2018)	Timor Leste	RCT (randomized controlled trial)	130	1–5 years	Ascariasis	26/130 (20%)	61.5%	[28]
					Hookworm infection	14/130 (10.8%)		
Ulayya et al. (2018)	Indonesia	Cross sectional	50	2–5 years	Helminthiasis (according to symptoms)	3/50 (6%)	26%	[29]

Jimenez et al. (2019)	Mexico	Cross sectional	178	0–5 years	Ascariasis	48/178 (27%)	41.8%	[30]
					Trichuriasis	6/178 (3.4%)		

Huus et al. (2020)	Madagascar and Central African Republic	Cross sectional	138	2–5 years	Helminthiasis	70/125 (56%)	51.4%	[31]

Yoseph and Beyene (2020)	Ethiopia	Cross sectional	622	6–59 months	Ascariasis	67/622 (10.8%)	39.3%	[32]
					Trichuriasis	38/622 (6.1%)		
					Hookworm infection	49/622 (7.9%)		
					Strongyloidiasis	10/622 (1.6%)		

Osman et al. (2020)	Ethiopia	Cross sectional	387	1–5 years	Ascariasis	57/387 (14.7%)	30%	[33]
					Trichuriasis	2/387 (0.5%)		
					Hookworm infection	6/387 (1.6%)		

**Table 3:** Summary of clinical manifestations in various studies.

Type of disease	Clinical manifestations	Effect size	*P*-value^*∗*^	Ref
Helminthiasis	Abdominal pain	*X* ^2^ = 2.63	0.105	[21]
	Abdominal bloating	*X* ^2^ = 11.84	0.001	
	Watery diarrhea	*X* ^2^ = 13.96	<0.001	
	Bloody diarrhea	*X* ^2^ = 1.52	0.217	
	Hepatomegaly	*X* ^2^ = 0.01	0.948	
	Anemic conjunctiva	*X* ^2^ = 3.44	0.064	
	Big belly	*X* ^2^ = 20.56	<0.001	
	Splenomegaly	*X* ^2^ = 0.79	0.373	
	Abdominal tenderness	*X* ^2^ = 2.29	0.130	
Hookworm infection	Pale subconjunctiva	aOR^a^ = 1.91		

Ascariasis/Trichuriasis coinfection	Iron deficiency anemia	aPR^b^ = 3.1		[22]

^a^Adjusted odds ratio. ^b^Adjusted prevalence ratio. ^*∗*^The test used was the chi-square test.

**Table 4:** Summary of laboratory findings in various studies.

Type of disease	Laboratory Findings	Effect Size	*P*-value	Ref
Stunting	Alpha 1-antitrypsin median (*μ*g/g):	*β* ^a^ = 0.41	0.044	[27]
	Stunting = 236.6			
	Normal = 151.4			
	The proportion of IgA-coated bacteria in feces is higher.	N/A	0.029	[31]

Ascariasis	Retinol binding protein <0.7 mg/L	aPR^b^ = 2.20		[22]
	Geometric mean EPG (eggs per Gram):			[17]
	7–9 months = 1.1			
	12–14 months = 2.4			
	Geometric mean EPG = 2.2			[24]
	EPG is higher in stunted children under five years of age.	IRR^c^ = 0.15	<0.001	[20]

Trichuriasis	Geometric mean EPG:			[17]
	7–9 months = 1.1			
	12–14 months = 3.1			
	Geometric mean EPG = 1.3			[24]

Hookworm infection	EPG is higher in the second infection in stunted children under five years of age.	IRR = 0.49	<0.06	[20]
	Geometric mean EPG:			[17]
	7–9 months = 1.1			
	12–14 months = 1.0			
	Geometric mean EPG = 1.0			[24]

Ascariasis/Trichuriasis coinfection	Ferritin <12 mg/L	aPR = 3.28		[22]

Environmental enteropathy	The intestinal inflammatory biomarker myeloperoxidase decreased as the age increased.	Coefficient: −0.29	0.002	[28]

^a^Regression coefficient. ^b^Adjusted prevalence ratio. ^c^Incidence rate ratio.

**Table 5:** Summary of risk factors for STH infection in various studies.

Author (Year)	Population Number	Prevalence	Risk Factor	Effect Size	*P*-value	Ref
Kounnavong et al. (2011)	570	215/570 (37.7%)	Socioeconomic status	AL^a^ OR^b^ = 0.61	0.043	[18]
			Age ≥48 months	HW^c^ OR = 1.75	0.041	

Jimenez et al. (2013)	250	93/250 (37.2%)	2–5 years of age^*∗*^	Pantepec = 17.737	Pantepek = 0.000	[19]
				Chanal = 11.942	Chanal = 0.001	
				Larrainzar = 7.343	Larrainzar = 0.007	

Cabada et al. (2014)	62	24/62 (38.7%)	Malnutrition^*∗*^	N/A	0.2	[23]

Jimenez et al. (2019)	178	Al: 38/74 (51.3%)	Stunting	AL OR = 9.81	<0.001	[30]

Yoseph and Beyene (2020)	622	164/622 (26.4%)	Stunting^*∗*^	N/A	AL and HW=<0.001	[32]
					TT^d^ = 0.007	
					SS^e^ = 0.22	
			Low wealth status	aOR^f^ = 1.04	>0.05	
			Drinking from unprotected water sources	aOR = 1.14	>0.05	
			Consumption of raw vegetables	aOR = 2.65	<0.05	
			Lack of sanitation facilities	aOR = 2.9	<0.01	
			Not wearing shoes	aOR = 3.5	<0.01	
			High number of family members	aOR = 2.7	<0.01	

^a^ *Ascaris lumbricoides.* ^b^Odds ratio. ^c^Hookworm. ^d^*Trichuris trichiura*. ^e^*Strongyloides stercoralis.*^f^Adjusted odds ratio. ^*∗*^Chi-square test.

## Data Availability

The data supporting this literature review were obtained from previously reported studies and datasets, which have been cited.
